# Characterization of *Paenibacillus larvae* bacteriophages and their genomic relationships to firmicute bacteriophages

**DOI:** 10.1186/1471-2164-15-745

**Published:** 2014-08-30

**Authors:** Bryan D Merrill, Julianne H Grose, Donald P Breakwell, Sandra H Burnett

**Affiliations:** Microbiology and Molecular Biology Department, Brigham Young University, Provo, UT USA

**Keywords:** *Paenibacillus larvae*, Bacteriophage, Jimmer1, Jimmer2, Emery, Abouo, Davies, phiIBB_Pl23, Phamerator, Phage genomics

## Abstract

**Background:**

*Paenibacillus larvae* is a Firmicute bacterium that causes American Foulbrood, a lethal disease in honeybees and is a major source of global agricultural losses. Although *P. larvae* phages were isolated prior to 2013, no full genome sequences of *P. larvae* bacteriophages were published or analyzed. This report includes an in-depth analysis of the structure, genomes, and relatedness of *P. larvae* myoviruses Abouo, Davis, Emery, Jimmer1, Jimmer2, and siphovirus phiIBB_Pl23 to each other and to other known phages.

**Results:**

*P. larvae* phages Abouo, Davies, Emery, Jimmer1, and Jimmer2 are myoviruses with ~50 kbp genomes. The six *P. larvae* phages form three distinct groups by dotplot analysis. An annotated linear genome map of these six phages displays important identifiable genes and demonstrates the relationship between phages. Sixty phage assembly or structural protein genes and 133 regulatory or other non-structural protein genes were identifiable among the six *P. larvae* phages. Jimmer1, Jimmer2, and Davies formed stable lysogens resistant to superinfection by genetically similar phages. The correlation between tape measure protein gene length and phage tail length allowed identification of co-isolated phages Emery and Abouo in electron micrographs. A Phamerator database was assembled with the *P. larvae* phage genomes and 107 genomes of Firmicute-infecting phages, including 71 *Bacillus* phages. Phamerator identified conserved domains in 1,501 of 6,181 phamilies (only 24.3%) encoded by genes in the database and revealed that *P. larvae* phage genomes shared at least one phamily with 72 of the 107 other phages. The phamily relationship of large terminase proteins was used to indicate putative DNA packaging strategies. Analyses from CoreGenes, Phamerator, and electron micrograph measurements indicated Jimmer1, Jimmer2, Abouo and Davies were related to phages phiC2, EJ-1, KC5a, and AQ113, which are small-genome myoviruses that infect *Streptococcus*, *Lactobacillus*, and *Clostridium,* respectively.

**Conclusions:**

This paper represents the first comparison of phage genomes in the *Paenibacillus* genus and the first organization of *P. larvae* phages based on sequence and structure. This analysis provides an important contribution to the field of bacteriophage genomics by serving as a foundation on which to build an understanding of the natural predators of *P. larvae*.

**Electronic supplementary material:**

The online version of this article (doi:10.1186/1471-2164-15-745) contains supplementary material, which is available to authorized users.

## Background

*Paenibacillus larvae (P. larvae)* is a sporulating Firmicute. It is the causative agent of American Foulbrood (AFB), a disease that infects and destroys the larvae of honeybees (*Apis mellifera*). The first eight *P. larvae* phages were reported between 1955 and 1999 and included BLA [1], L3 [2], BL2 [3], PBL1 [4], PBL0.5 [5], PBL2 [5], PBL3 [6], and PPL1c [7]. These phages have not been sequenced. Most of these phages were isolated from lysogens and were used to characterize different strains of *P. larvae* [8–10]. Phages that infect *P. larvae* were originally identified as *Bacillus larvae* phages; however, the names of these phages were changed to *Paenibacillus larvae* phages following the reclassification of the bacteria [11, 12].

Recent advances in DNA sequencing technology have made it possible to sequence many bacteriophage genomes. When these sequences are analyzed, putative protein functions can be determined. Other studies have used comparative genomics to organize phages into related clusters [13], correlate phage packaging mechanisms with large terminase protein sequences [14], and study gene transfer, phylogenetic relationships, and impacts on host virulence [15, 16].

Comparative genomics can be accomplished using software specialized for phage genomes such as the computer program, Phamerator [17]. Phamerator incorporates available data for each genome entered into its database, such as bacterial host, annotations of genes from GenBank, and conserved domains [18]. Phamerator compares each gene product in the database to each other using BLASTP [19] and ClustalO [20], the scores of which are used to create phamilies (phams) of related gene products. Phamerator provides visual tools such as full genome comparison maps and can display the relationships between proteins within a pham using a circular diagram (pham circle). Proteins within each pham must meet or exceed user-defined cutoffs for E-values and percent identity for at least one other gene product in the pham. Strict cutoffs result in phams that indicate a shared similar function and predict phylogenetic relationships.

In 2013, six *P. larvae* phages were isolated. These phages were fully sequenced and their genomes published [21, 22]. *P. larvae* siphovirus phiIBB_Pl23 was isolated in Portugal [21] and *P. larvae* myoviruses Abouo, Davies, Emery, Jimmer1, and Jimmer2 were isolated in Utah [22]. In this report we compare the genomes of the six fully-sequenced *P. larvae* phages, categorize all published *P. larvae* phages into three groups based on structural morphology, use Phamerator to analyze previously unexplored *Paenibacillus* phages, and explore genetic relationships of the *P. larvae* phages with 107 other phages that infect Firmicute hosts. We identify gene products with conserved domains including a putative bacteriocin, serine recombinase, and antirepressor, and investigate their conservation among *P. larvae* phages. Results from Phamerator and CoreGenes indicate a relationship between four *P. larvae* phages and four small genome myoviruses that infect *Lactobacillus*, *Clostridium* and *Streptococcus*. These results show that comparisons can be drawn between phages that infect a phylum and provide a basis for analyzing and comparing newly isolated phages that infect *P. larvae*.

## Results

### Bacterial identification, phage isolation, and phage sequencing

Bacterial isolates were collected from spores found in honey samples. All characteristic tests for *P. larvae* were positive: the isolates grew on PLA plates and were catalase negative and Gram-positive. PCR products from 16S rRNA primers were sequenced using BigDye sequencing. BLAST results from nine of the ten 16S rRNA sequences showed more than 99% similarity with *Paenibacillus larvae subsp. pulvifaciens* strains DSM 8442 and DSM 8443 as well as the related bacteria *Brevibacillus laterosporus*. Of nine isolates, PL2 and PL6 were used for phage isolation. Phages Abouo, Davies, and Emery were isolated using PL6, while phages Jimmer1 and Jimmer2 were isolated using PL2.

Each *P. larvae* phage sample was plaque-purified at least three times, sequenced, and published [22]. Prior to genome sequencing and electron microscopy we were unaware that one phage sample still contained two different phages. Plaque purification did not successfully separate these two phages. However, assembly of the genomes revealed two clearly independent genomes that separated with ease with over 100-fold coverage of the genomes. These co-isolated phages were named Emery and Abouo. Results from sequencing and annotation of the six *P. larvae* phages are found in Table 1. The genomes varied in length from ~40 kb to ~58 kb. Most of the genes in each genome were located on the forward strand (90% ± 3%). The average G + C content for these phages is 39.48% ± 1.41%. BLAST hits for proteins within these phages included both *Paenibacillus* and *Brevibacillus* bacterial strains as well as bacteriophages that infected other Firmicutes.

**Table 1:** **Characteristics of sequenced** ***Paenibacillus larvae*** **bacteriophages**

***P. larvae***phage	Host strain of ***P. larvae***	Accession number	Genome length	Number of genes	Forward genes	Reverse genes	GC content
**Abouo**	PL6	KC595517	45552 bp	102	86	8	39.16%
**Davies**	PL6	KC595518	45798 bp	94	84	10	39.10%
**Emery**	PL6	KC595516	58572 bp	94	87	15	41.44%
**Jimmer1**	PL2	KC595515	54312 bp	102	91	11	38.11%
**Jimmer2**	PL2	KC595514	54312 bp	102	91	11	38.10%
phiIBB_Pl23 *	H23	KF010834	41294 bp	68	63	5	40.94%

*Paenibacillus phage phiIBB_Pl23 data from [21].

### Phage cross-infectivity, lysogeny, and lysogen superinfection

Plaques from the sample containing Emery and Abouo were clear while plaques from the other three phages were hazy. Phages Jimmer1 and Jimmer2 were isolated independently using PL2 and neither of these phages could infect PL6. Phages Davies and Emery/Abouo were isolated using PL6 and these phages could not infect PL2. None of these phages were able to produce plaques on lawns of *B. cereus, B. subtilis, L. acidophilus, S. aureus, or S. epidermidis*. Of the four phage samples, three formed stable lysogens: Jimmer1 in PL2, Jimmer2 in PL2, and Davies in PL6. Stable lysogens were identified when phages were not able to superinfect bacteria lysogenic for the same phage. The lysate containing Emery and Abouo induced lysis in the PL6 Davies lysogen. No other superinfection or induced lysis was observed in any other lysogen-phage combinations. The sample containing Emery and Abouo did not form a stable lysogen and no superinfection data were obtained.

### Electron microscopy reveals myovirus structure for five *P. larvae*phages

**Figure 1:**
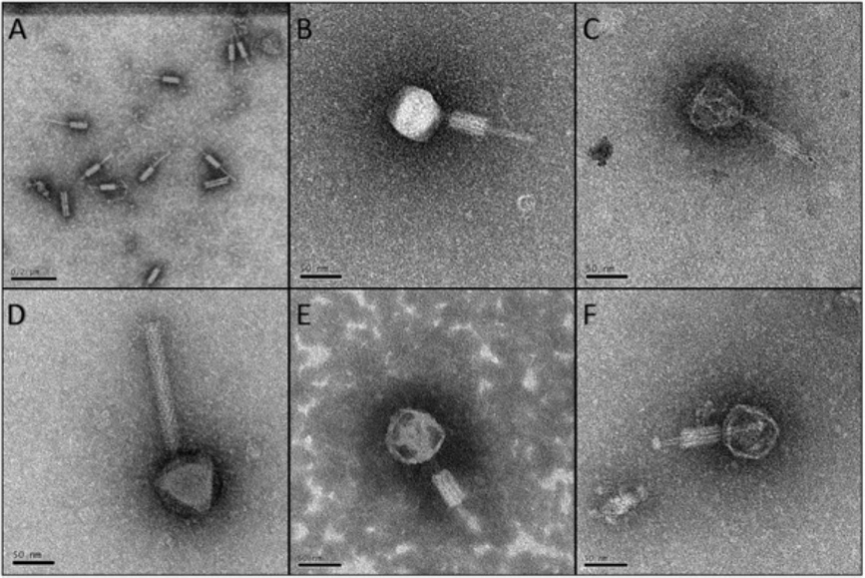
**Electron micrographs of** ***P. larvae*** **phages. A)** Tails and tail sheaths of *P. larvae* phages separated from the capsids (Jimmer1). These structures were more abundant than intact phages in electron micrographs. Scale bar represents 0.2 μm. **B)** Phage Abouo. **C)** Phage Davies. **D)** Phage Emery. **E)** Phage Jimmer1. **F)** Phage Jimmer2. Scale bars represent 50 nm for panels **B**-**F**.

Electron microscopy revealed that these five *P. larvae* phages were myoviruses, marked by the presence of a contractile tail sheath (Figure 1). Figure 1A shows tail structures separated from the phage capsids. These tail structures were more abundant than intact phages in all samples submitted for electron microscopy. Figure 1B, 1C, 1E, and 1F show intact phages with contracted tail sheaths, while Figure 1D shows an extended tail sheath. Because phages Emery and Abouo were not separated, micrographs for these phages were taken from the same copper grid.

### Sequence similarities between phages

**Figure 2:**
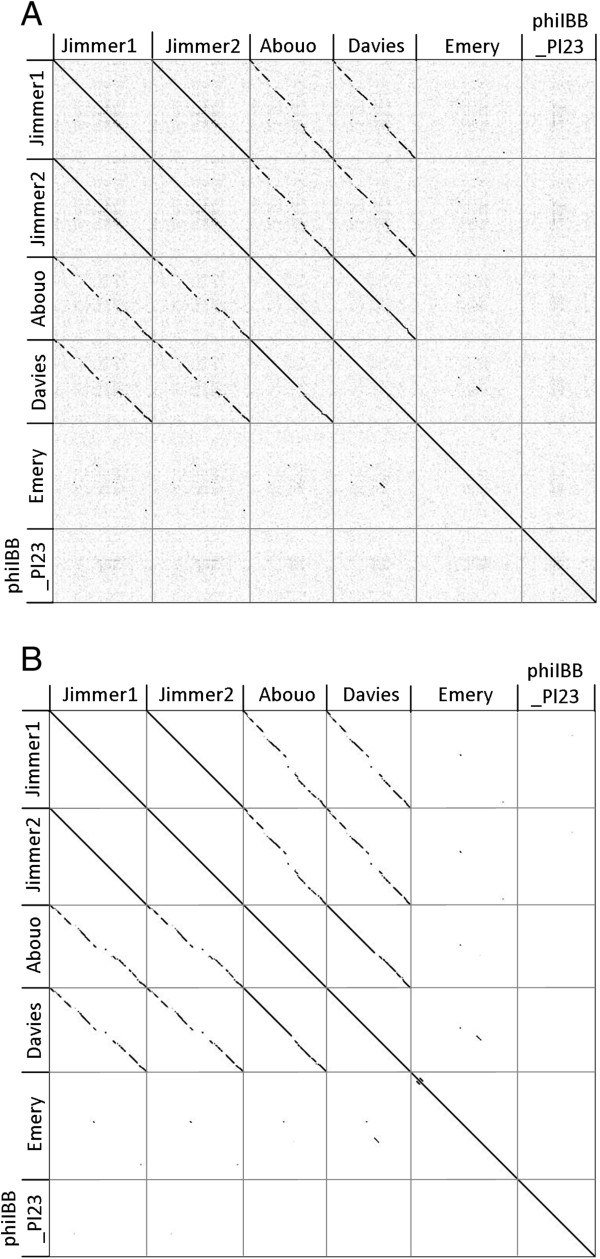
**Dotplots of six** ***P. larvae*** **phage genomes. A)** Nucleic acid comparison of full genomes. **B)** Amino acid comparison of coding regions in each genome.

Gepard dotplots of the full genome sequences for all six *P. larvae* phages are shown in Figure 2. Diagonal lines within the dotplots indicated that phages Abouo, Davies, Jimmer1, and Jimmer2 were very similar to each other. Host specificity was also reflected in similarities between these four phages. For instance, the PL2 phages Jimmer1 and Jimmer2 shared 99.8% average nucleotide identity and the PL6 phages Abouo and Davies shared 94.9% identity. However, the average nucleotide identity between PL2 phages Jimmer1 and Jimmer2 and PL6 phages Abouo and Davies was only 80.5%. The lack of diagonal lines in the dotplot indicated that phages Emery and phiIBB_Pl23 were very different from the other *P. larvae* phages examined. While the Emery and Abouo phages were found in the same sample, sequences assembled independently without conflict with over 100-fold coverage for each genome [22]. No similarities between the sequences of Emery and Abouo were apparent in the dotplot (Figure 2). However, a black line indicates a small section of homology between Emery and Davies that Davies does not share with Abouo. Because Emery and Abouo were co-isolated and sequenced together, individual reads in these sections of Emery, Davies, and Abouo were scrutinized using Consed [23] to ensure the assemblies were correct. The fold coverage before, throughout, and after these sections of Emery, Davies, and Abouo was at least 80.

### Distinguishing between phages Emery and Abouo

In addition to the separation of unique DNA sequences for Emery and Abouo in the same sample, two markedly different phages were present in electron micrographs based on measurements of capsid height and tail length. Putative tape measure protein (TMP) genes were identified in each phage genome. The TMP gene found in Emery (gp16) was 3,000 bp long, while the TMP gene in Abouo (gp20) was 2,055 bp long, showing that the TMP gene in Emery was 1.46 times longer than the TMP gene in Abouo. The positive correlation between TMP gene length and tail length [24] was used to suggest a correlation between phages Emery and Abouo in electron micrographs and their respective genome sequences. The average tail length for Emery was 162.2 nm long while the average tail length for Abouo was 113.6 nm, making the tail of phage Emery 1.43 times longer than the tail of phage Abouo (Table 2). In comparison, the TMP gene of Emery was 1.46 times longer than the TMP gene in Abouo. Based on this data, we matched the long TMP gene and tail length with Emery, and the short TMP gene and tail length with Abouo (Figure 1).

**Table 2:** **Comparison of** ***Paenibacillus larvae*** **phage structures from electron microscopy data**

Phage name	Place isolated	Capsid height (nm)	Capsid width (nm)	Tail length (nm)	Tail width (nm)	Contracted sheath length (nm)	Contracted sheath width (nm)
***Myoviridae***							
Abouo	Utah, USA	68.3 ± 5.6	65.1 ± 3.6	113.6 ± 2.6	12.7 ± 3.8	45.7 ± 5.0	24.0 ± 2.3
Davies	Utah, USA	68.1 ± 4.5	61.7 ± 3.2	125.4 ± 0.2	9.8 ± 0.7	83.0 ± 0	19.2 ± 0
Emery	Utah, USA	73.5 ± 3.2	66.0 ± 3.3	162.2 ± 5.2	11.0 ± 1.4	82.9 ± 0	25.5 ± 0
Jimmer1	Utah, USA	68.2 ± 3.6	61.3 ± 3.9	98.9 ± 14.7	11.9 ± 1.4	43.1 ± 1.5	23.3 ± 2.5
Jimmer2	Utah, USA	62.9 ± 2.5	66.3 ± 2.2	75.6 ± 8.7	7.7 ± 0.3	42.9 ± 2.4	20.6 ± 0.4
PBL0.5c* (and PBL2 *)	Georgia (Minnesota)	66.3 ± 3	66.3 ± 3	142.0 ± 1	9.2 ± 0.9	92.0 ± 1	22.5 ± 1.2
BL2*	Unknown	64.1 ± 1.1**	60.1 ± 3.9**	116	7	76	19
***Siphoviridae***							
phiIBB_Pl23*	Portugal	Not published					
***Round capsid***							
PBL3*	USA	62.2 ± 2.2**	70 ± 2.1	140 ± 2.8	10.1 ± 0.9	—	—
***Elongated capsid***							
BLA*	Czechoslovakia	150	68	280	13	—	—
PBL1* (and PPL1c *)	USA	95.9 ± 2.6	52.5 ± 1.9	131.8 ± 1.5	9.4 ± 0.6	—	—

*Data for *Paenibacillus larvae* phages BLA, BL2, PBL1, PBL2, PBL0.5c, PBL3, PPL1c, and phiIBB_Pl23 were taken from referenced publications [1, 3–7, 21, 22]. Independent electron microscopy data for phages PBL2 and PPL1c were not published; instead, they were reported to be indistinguishable from PBL0.5c and PBL1, respectively.

**Measurements were taken from published electron micrographs instead of reported data which were either not presented or were inaccurate.

— Phage measurements were not taken.

### Measurements from electron micrographs

*P. larvae* phages were separated into three distinct groups based on structural morphology. The first group of phages were myoviruses (icosahedral capsids and contractile tails) and included phages Abouo, Davies, Emery, Jimmer1, Jimmer2, PBL0.5 [5], and PBL2 [5]. There were two distinct groups of phages that were siphoviruses. The first siphovirus group contains phages BLA [1], PBL1 [4], and PPL1c [7]. These phages had long, non-contractile tails and elongated capsids. The second siphovirus group contained only PBL3 which had a round capsid [6]. Phage phiIBB_Pl23 was also a siphovirus [21], but could not be categorized into one of the two siphovirus groups because images or measurements were not yet available. There was no apparent correlation between the type of phage and where the phage was isolated. Measurements taken from all published electron micrographs of *P. larvae* phages are shown in Table 2. Measurements for phages Abouo, Davies, Emery, Jimmer1, and Jimmer2 were taken from at least three different intact phages. Phages grouped into categories by morphology type were found to have similar structural measurements. The structures of the *P. larvae* myoviruses were similar in size with an average capsid height of 67.2 ± 3.2 nm and an average width of 64.1 ± 2.6 nm. The average tail length was 122.0 ± 27.3 nm and was the most variable of the measured phage features.

### Frameshift in *P. larvae*phage Emery

Phage Emery exhibited a putative ribosomal slippage site in gp4 that encoded for a head morphogenesis protein in the SPP1 gp7 family. This frameshift was identified by the online frameshift finding tool FrameD [25]. The two products in Emery are predicted to be 82.5 kDa following ribosomal slippage and 58.9 kDa if there is no slippage. The presence of both head morphogenesis proteins in the Emery virion has not yet been verified. We were unable to detect a putative frameshift via FrameD in *Bacillus* phage SPP1 or any protein sequence homology using BLASTP between the morphogenesis proteins in Emery and SPP1.

### *P. larvae*phage genomic comparison using Phamerator software

A database of phage genomes related to the *P. larvae* phages was assembled for analysis using the phage genome comparison program Phamerator [17]. The finished Phamerator database contained a diverse set of phages that infected Firmicute bacteria including the 6 *P. larvae* phage genomes, 71 *Bacillus* phages, 1 *Clostridium* phage, 3 *Enterococcus* phages, 2 *Geobacillus* phages, 7 *Lactobacillus* phages, 6 *Listeria* phages, one *Paenibacillus glucanolyticus* phage, 15 *Staphylococcus* phages, and 1 *Streptococcus* phage (Additional file 1). Phages included in the database were selected based on BLAST hits to gene products identified during annotation of the *P. larvae* phage genomes. Phamerator grouped the 13,697 putative proteins annotated in the 113 phage genomes into 6,181 phamilies (phams). Only 2,233 phams (36.1%) contained two or more members. These 2,233 phams contained 9,749 (71.1%) of the 13,697 putative proteins in the database. The remaining 3,948 (28.8%) putative proteins could not be grouped with other proteins and were designated as "orphams" [17].

The Phamerator database allowed comparison of *P. larvae* phage genes to each other and to phages infecting other bacteria. A spreadsheet was exported from the Phamerator database to report all phage gene products in the database, the phams to which the gene products are assigned, and the conserved domains found in gene products in those phams (Additional file 2). Table 3 indicates how many putative proteins in each *P. larvae* phage are orphams, are shared only with *P. larvae* phages, or are shared with phages infecting other bacterial hosts. Putative proteins from Jimmer1 and Jimmer2 shared phams with 56 non-*P. larvae* phages, while Abouo shared phams with 52, Davies with 53, Emery with 24, and philBB_P123 with 57 other non-*P. larvae* phages. Between the six *P. larvae* phages, there are phams shared with 72 non-*P. larvae* phages of the 107 (67.3%) included in the database. Of 562 genes in the six *P. larvae* phages, only 114 (20.3%) encoded proteins grouped into phams with proteins from other types of phages in the database. Of the remaining genes, 300 (53.4%) encoded proteins that were grouped into phams containing only *P. larvae* phage proteins and 148 (26.3%) were orphams.

**Table 3:** **Comparison of** ***P. larvae*** **phage genes**

Phage name	Genes in pham with non-PL phage	Genes only in PL phages	Orphams	# of genes	# of genes with BLAST* hits (%)	# of genes with BLAST* functions (%)	# of genes with CD (%)
Abouo	24	63	7	94	86 (91%)	47 (50%)	41 (44%)
Davies	23	67	4	94	85 (90%)	48 (51%)	42 (45%)
Emery	12	9	81	102	74 (73%)	40 (39%)	33 (32%)
Jimmer1	22	80**	0	102	92 (90%)	50 (49%)	50 (49%)
Jimmer2	22	80**	0	102	92 (90%)	50 (49%)	50 (49%)
phiIBB_Pl23	11	1	56	68	55 (81%)	32 (47%)	27 (39%)
All *P. larvae* phages	114	300	148	562	484 (86%)	267 (48%)	243 (43%)

*BLAST hits had an E-value of less than 1 × 10^-4^.

**Of the 80 *P. larvae* phage genes in Jimmer1 and Jimmer2, 31 are not found in any other phages in the database.

Pham groupings reflected the genetic relationships of *P. larvae* phages. The genomic sequence comparison of Jimmer1 and Jimmer2 using ClustalW identified differences in 80 bp of the 54,312 bp genomes (99.85% similar) [22]. All corresponding genes between Jimmer1 and Jimmer2 shared the same phams. Of the 80 *P. larvae* phage genes in Jimmer1 and Jimmer2, 31 were unique to these two phages and were not found in any other phages in the database. These 31 genes would be orphams if Jimmer2 were not isolated (Table 3).

Phamerator identified conserved domains in at least one gene in 1,501 phams (24.3%) of the total 6,181 phams in the database. Although many *P. larvae* phage genes encoded proteins with significant BLAST hits, less than half of the proteins had a known function. Of all *P. larvae* phage putative proteins, 86% had a BLAST hit with an E-value less than 1 × 10^-4^ (see Table 3), yet only 48% of the proteins returned BLAST hits listing a function. Conserved domains were identified in only 43% of the *P. larvae* phage putative proteins (Table 3). Phages Emery and phiIBB_Pl23 contained the most orphams, the fewest BLAST hits, and the most putative proteins with no identifiable conserved domains.

### *P. larvae*phages share structural and regulatory genes with similar functions

Conserved domains and BLAST hits matching phage or bacterial proteins were used to assign functions to 234 gene products in the six *P. larvae* phages, indicating that these genes were not novel and were characteristically found in other phages. The assembly and structural proteins were grouped according to function in Table 4. The regulatory and non-structural proteins are listed in Table 5. The pham assignment for each gene is shown in parentheses. Pham numbers are specific to the Phamerator database used for this analysis.

**Table 4:** ***P. larvae*** **phage assembly proteins and structural proteins; gene product # and (pham #, specific to this analysis)**

Function	Abouo	Davies	Jimmer1 and 2	Emery	phiIBB_Pl23
Terminase small subunit	*gp1 (1)*	*gp1 (1)*	*gp1 (1)*		gp1 (656)
Terminase large subunit	*gp2 (2)*	*gp2 (2)*	*gp2 (2)*	gp3 (5610)	gp2 (399)
Phage portal protein	*gp3 (3)*	*gp3 (3)*	*gp4 (3)*		gp3 (657)
SPP1 Gp7 family head morphogenesis protein	*gp4 (4)*	*gp4 (4)*	*gp5 (4)*	gp4 and 5 (5611)	
Phage virion morphogenesis family protein				gp10 (5616)	
Phage minor structural GP20 family protein	*gp5 (5)*	*gp5 (5)*	*gp6 (5)*		
Prohead core scaffolding/protease				gp6 (5612)	
Phage major capsid protein E	gp7 (7)	gp7 (7)	gp8 (3028)		gp5 (659)
DNA packaging protein	*gp9 (9)*	*gp9 (9)*	*gp10 (9)*		
Head-tail joining protein	*gp10 (10)*	*gp10 (10)*	*gp11 (10)*		gp7 (5912), gp8 (2096)
Phage tail sheath protein	*gp14 (14)*	*gp14 (14)*	*gp15 (14)*		
Tail length tape measure protein	*gp20 (20)*	*gp20 (20)*	*gp24 (20)*	gp16 (5622)	gp14 (397)
Baseplate J family protein	*gp27 (27)*	*gp27 (27)*	*gp29 (27)*	gp21 (5627)	
Tail fiber	gp30 (30), gp31 (31)	gp30 (30), gp31 (31)		gp24 5630)	
Tail protein	*gp15 (15)*	*gp15 (15)*	*gp16 (15)*	gp12 (5618), gp18 (5624)	gp10 (5914), gp11 (5915), gp15 (5918), gp17 (5920)

**Table 5:** **Regulatory proteins and other non-structural proteins; gene product # and (pham #, specific to this analysis)**

Function	Abouo	Davies	Jimmer1 and Jimmer2	Emery	phiIBB_Pl23
LysM domain-containing protein (peptidoglycan binding)	*gp22 (22)*	*gp22 (22)*	*gp25 (22)*	gp17 (5623)	
Cell wall hydrolase/late control D	*gp23 (23)*	*gp23 (23)*	*gp26 (23)*		
Subtilisin-like serine protease			gp42 (3042)		
Peptidoglycan hydrolase	*gp36 (36)*	*gp36 (36)*	*gp38 (36)*	gp31 (5634)	gp21 (351)
Beta-lactamase					gp47 (5949)
Holin					gp22 (5924)
Phage-like element PBSX protein	*gp26 (26), gp28 (28)*	*gp26 (26), gp28 (28)*	*gp28 (26), gp30 (28)*	gp22 (5628)	
bhlA/Bacteriocin	*gp34 (34)*	*gp34 (34)*	*gp36 (34)*	gp29 (34)	gp20 (5923)
Membrane protein	gp35 (35), gp44 (44)	gp35 (35), gp44 and gp45 (44)		gp34 (5637), gp53 and gp54 (44)	
Antirepressor	gp41 (41), *gp68 (71)*	gp41 (41), *gp72 (71)*	gp19 (951), *gp79 (71)*		gp39 (951)
Tyrosine Recombinase XerC	gp43 (43)	gp43 (43)		gp1 (5608), gp47 (43)	
Accessory gene regulator B family protein	gp45 (45)	gp46 (45)		gp55 (45)	
AbrB family transcriptional regulator				gp72 (2041)	
RuvC Holliday junction resolvase					gp59 (5961)
ArpU family transcriptional regulator					gp61 (2944)
ABC transporter-like protein	*gp74 (76)*	*gp77 (76)*	*gp84 (76)*		gp23 (5925)
Arc-like DNA binding domain	*gp52 (53)*	*gp54 (53)*	*gp62 and gp63 (53)*		
Single-stranded DNA-binding protein	*gp62 (65), gp63 (66)*	*gp66 (65), gp67 (66)*	*gp73 (65), gp74 (66)*		
HNH endonuclease	*gp64 (67)*	*gp68 (67)*	*gp75 (67)*		gp64A (5966)
Serine recombinase			gp49 (3048)		gp33 (3048)
SOS-response repressor and protease LexA					gp31 (5934)
Recombinational DNA repair protein RecT					gp49 (5948)
Phage replication protein O			gp76 (3058)		gp52 (5951)
DNA replication protein	gp66 (69)	gp70 (69)	gp77 (3059)	gp99 (653)	gp50 (5952)
YopX protein	*gp71 (74)*	*gp75 (74)*	*gp82 (74)*		gp54 (5966)
Toxin 1					gp26 (5928)
Recombinase recU	*gp73 (75)*	*gp76 (75)*	*gp83 (75)*		
dUTPase	*gp76 (78)*	*gp79 (78)*	*gp86 (78)*		
Site-specific DNA methylase	gp79 (3134)	gp81 (80)	gp88 (80)	gp33 (5636)	
RNA polymerase sigma-70 factor	*gp91 (90)*	*gp91 (90)*	*gp99 (90)*	gp94 (5683)	
DNA-dependent DNA polymerase family A				gp81 (634)	
Virulence-associated E family protein				gp96 (651)	
Stage V sporulation protein K				gp95 (4849)	
VRR-NUC domain-containing protein				gp98 (652)	
Transcriptional regulator (HTH, XRE)	*gp49 (50), gp50 (51), gp51 (52)*, gp54 (57), *gp56 (59)*	*gp51 (50), gp52 (51), gp53 (52)*, gp56 (55), gp57 (56), gp58 (57), *gp60 (59)*	*gp52 and gp56 (50), gp57 (51), gp58 (52), gp67 (59),* gp18 (3031), gp17 (3030), gp65 (3057), gp60 (3054), gp61 (3055)	gp60 (5656), gp89 (5678), gp40 (2605), gp65 (2691), gp64 (2692), gp48 (5649), gp59 (5655), gp49 (5650)	gp32 (5935), gp55 (5957)

Most of the functions listed in Tables 4 and 5 describe proteins found in more than one phage. For example, all five of the *P. larvae* myoviruses contained seven proteins that belonged to the same family or superfamily but not always to the same pham. The function of these proteins includes head morphogenesis, tape measure, baseplate (see Table 4), LysM peptidoglycan binding, peptidoglycan hydrolase, PBSX, and bacteriocin (see Table 5).

Few proteins with known functions were identified as putative virulence factors. BLAST results indicate that gp26 in *P. larvae* phage phiIBB_Pl23 is a protein that is toxic to insect larvae. No toxin genes were identified in the *P. larvae* myoviruses. Other host-related proteins include an ABC transporter-like protein found in *P. larvae* phages Abouo, Davies, Jimmer1, Jimmer2, and phiIBB_Pl23 as well as an XRE-family transcriptional regulator found in all *P. larvae* phages. The five myoviruses contained between five and ten of these regulators per genome compared to only two in the siphovirus phiIBB_Pl23. Abouo gp51, Jimmer1 gp58, Jimmer2 gp58 and Emery gp40, gp64, and gp65 (Table 5) are the only transcriptional regulators that share a pham with a non-*P. larvae* phage. All others are only found in *P. larvae* phages. It is not known what effects these transcriptional regulators have on the host, but they do contain a canonical helix-turn-helix (HTH) domain. Very few of the regulatory genes in these phages have known functions.

### Phage genome organization and pham groupings indicate relatedness of four *P. larvae*phages

**Figure 3:**
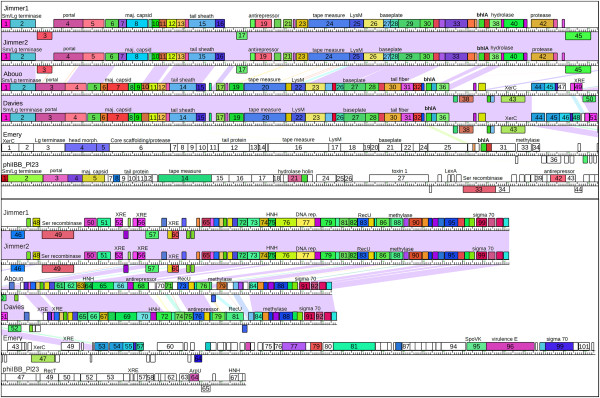
**Linear genome map of the six** ***P. larvae*** **phages.** Connecting lines between Jimmer1, Abouo, Davies, and Emery represent nucleotide similarity (E-value less than 1 × 10^-4^). Genes are color-coded according to phams of the encoded protein. Gene numbers are indicated inside gene boxes. Functions matching gene products described in Tables 4 and 5 are also displayed.

A linear genome map of the six *P. larvae* phages shows that the genes in phages Jimmer1, Jimmer2, Abouo, and Davies are organized similarly (Figure 3). Identically colored genes encode products that share a pham, while white genes encode orpham gene products. There are 58 phams that each contained gene products from phages Abouo, Davies, Jimmer1, and Jimmer2. Proteins in 30 of these phams had identifiable functions based on BLAST hits and are italicized in Tables 4 and 5. Of the 58 conserved phams, 38 did not contain homologs from any other phage in the database. Of the remaining 20 phams that have homologs from other phage types, three of the most populated phams are those containing small terminase (13 members), large terminase (14 members), and portal protein (13 members). Of the 16 other phages that shared one of these phams with the four similar *P. larvae* myoviruses, only three phages shared all three phams: Staphylococcus phages 37, 88, and PH15.

### Phamily relationships of large terminase proteins indicate putative DNA packaging strategies

**Figure 4:**
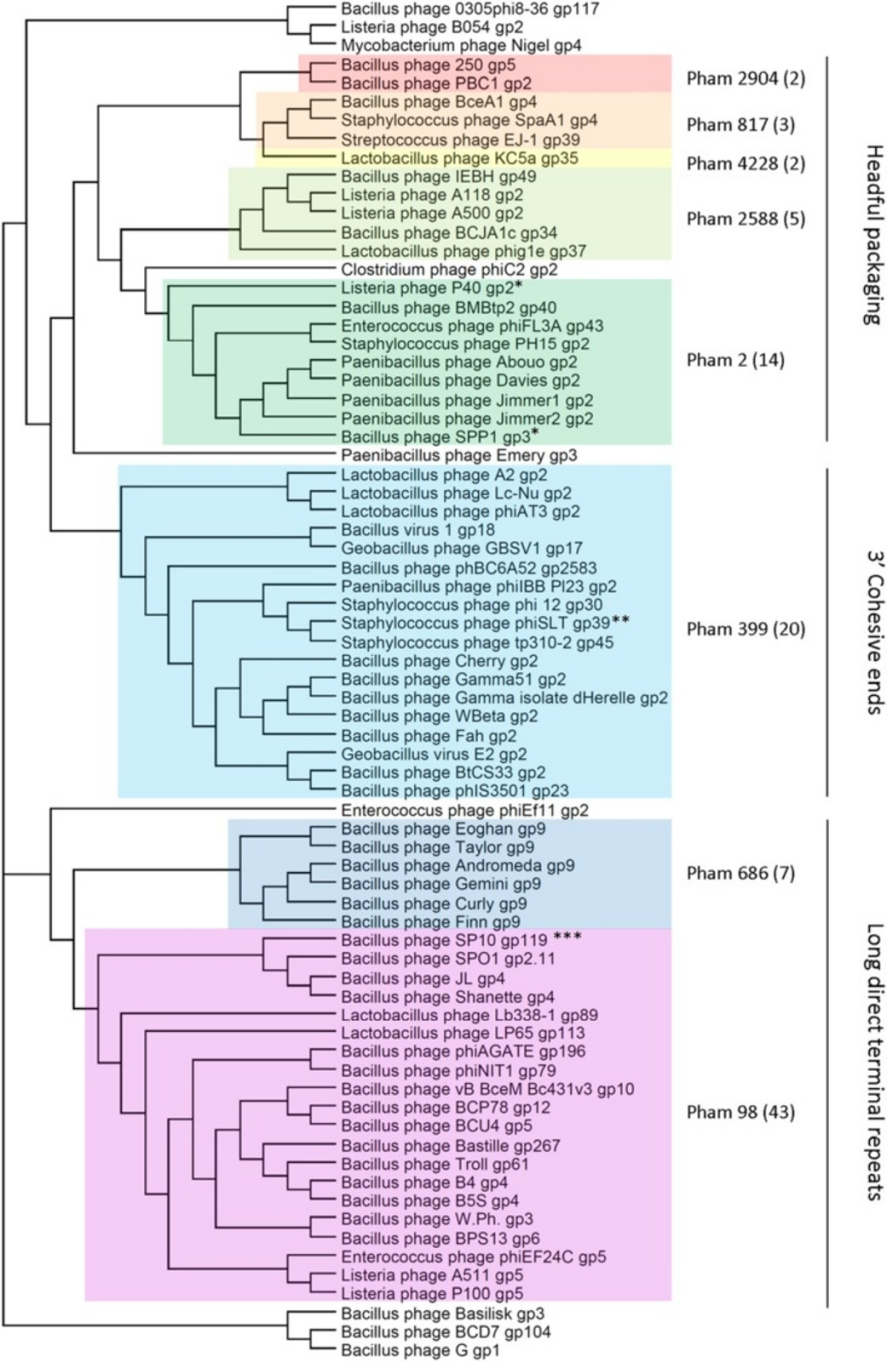
**Neighbor-joining phylogenetic tree of the large terminase gene products from the Phamerator database indicate proposed packaging strategies.** Colored boxes indicate proteins belonging to the same pham. Proteins that are not highlighted are orphams. Large terminase proteins grouped into similar phams are closely related on the tree and share a packaging strategy. *Experimentally determined headful packaging, circularly permuted terminal repeats [26, 27], **Experimentally determined 3′ cohesive ends [28]. ***Experimentally determined, long direct terminal repeats [29].

Phage gene products must meet stringent parameters in order be grouped into a pham with other genes that encode similar proteins. Because gene products in a pham are highly similar, phylogenetic analysis indicates that these proteins will be more closely related than others with the same function. A neighbor-joining phylogenetic tree grouped large terminase proteins in the Phamerator database by phamily (Figure 4). Amino acid sequences of large terminase proteins can indicate the DNA packaging strategy [14]. Phages Abouo, Davies, Jimmer1, and Jimmer2 likely use headful packaging and have circularly permuted terminal repeats based on close association with the large terminases of well-characterized headful packaging phages P40 [26], and SPP1 [27] which share a pham. Phage phiIBB_Pl23 likely has 3′ cohesive ends based on close association with phage phiSLT [28]. Further analysis of experimental data indicated that no phams generated by Phamerator contained terminases belonging to phages with different packaging strategies (data not shown). The packaging strategy for phage Emery is still undetermined because its large terminase protein is an orpham.

### *P. larvae*phages exhibit genetic and structural similarity with other small genome myoviruses.

The putative proteins encoded in *P. larvae* phages Abouo, Davies, Jimmer1, and Jimmer2 were often grouped into phams with proteins in *Clostridium* phage phiC2, *Lactobacillus* phage KC5a, and *Streptococcus* phage EJ-1. The similar proteins were mostly structural (Table 6) and included the terminase (small subunit), portal, head morphogenesis, minor structural, tail sheath, and baseplate proteins. All of the gene products listed in the table were grouped into the same pham except for three proteins that narrowly missed the pham cutoff values and are marked by asterisks. The tape measure proteins in Abouo, Davies, Jimmer1, and Jimmer2 were somewhat similar to those found in Streptococcus phage EJ-1 (average E-value < 9 × 10^-17^, average identity = 24%) and Clostridium phage phiC2 (average E-value < 8 × 10^-17^, average identity = 22%) but were not near the pham cutoff values of 1 × 10^-50^.

**Table 6:** **Genetic comparison of small genome myovirus phams and gene products within each pham**

Function	Pham number	Phages Abouo, Davies	Phages Jimmer1, Jimmer2	Clostridium phage PhiC2	Lactobacillus phage KC5a	Streptococcus phage EJ-1
Terminase (small subunit)	1	gp1	gp1		gp34	
Portal protein	3	gp3	gp4	gp3	gp36	gp40
Head morphogenesis protein	4	gp4	gp5	gp4	(gp37)*	(gp41)**
Minor structural protein	5	gp5	gp6		gp38	(gp46)***
Tail sheath protein	14	gp14	gp15	gp13	gp45	gp53
Baseplate J family protein	27	gp27	gp29	gp25	gp53	gp61
XRE family transcriptional regulator	52	gp51, 53	gp58		gp5	
Arc-like DNA-binding protein	53	gp52, 54	gp62, 63	gp53, 60		

No genes in Emery were grouped into any of the phams listed in this table. Genes followed by an asterisk were not grouped into the same phams as the *P. larvae* phages using Phamerator. The average E-value and percent identity when compared to Abouo, Davies, Jimmer1, and Jimmer2 is reported below:

*KC5a gp37 = 1 × 10^-46^, 31% identity.

**EJ-1 gp41 = 1 × 10^-39^, 31% identity.

***EJ-1 gp46 = 8 × 10^-14^, 32% identity.

The electron micrographs of *P. larvae* phages Abouo, Davies, Jimmer1, and Jimmer2 reveal a structure similar to those reported for *Clostridium* phages phiC2 [30], phiCD27 [31], and phiCD119 [32], *Lactobacillus* phages KC5a [33] which is reported as similar to *Lactobacillus* phages KC21T [33] and phiAQ113 [34], and *Streptococcus* phage EJ-1 [35]. The capsid of *Clostridium* phage phiC2 is 65 nm in diameter and the tail is 148 nm long. The capsid of *Streptococcus* phage EJ-1 is 57 nm in diameter and the tail is 130 nm long. The capsid of *Lactobacillus* phage KC5a was not measured, however it was reported as similar to KC21T, which had a capsid diameter of 45 nm and a tail length of 160 nm. KC5a was also reported to be genetically similar to phiAQ113, which has a capsid diameter of 55 nm, a tail length of 147 nm, and a tail sheath width of 22 nm. Phamerator grouped all KC5a gene products listed in Table 6 into the same phams as phiAQ113 genes except the small terminase (absent in phiAQ113) and the head morphogenesis protein.

Although *P. larvae* phage Emery contained gene products with the same functions as those listed in Table 6, the proteins were all orphams. However, the first five gene products encoded in *P. larvae* phage phiIBB_Pl23 (small terminase, large terminase, portal protein, protease, and major capsid proteins) all shared a pham with similar proteins from five siphovirus *Staphylococcus* phages (3A, 47, phi12, phiSLT, and tp310-2).

The program CoreGenes 3.5 was used to further compare the genes in the *P. larvae* phages with small-genome myoviruses. Using the default BLASTP threshold of 75, core proteins were identified in the five *P. larvae* myoviruses with respect to *Clostridium* phage phiCD119, *Streptococcus* phage EJ-1, *Lactobacillus* phage KC5a, and *Lactobacillus* phage AQ113. The number of core proteins shared between comparison and reference genomes are listed in Table 7. The percent of core proteins with respect to the reference genome are also reported. *Clostridium* phage phiCD119 was the only one of these phages that belonged to a genus (phiCD119likevirus); the other three are currently unclassified. Previous analyses of the *Podoviridae* and *Myoviridae* families grouped phages together when phages share 40% of core proteins with a reference phage genome [36, 37]. Based on this cutoff value, Abouo, Davies, Jimmer1 and Jimmer2 formed a new group of small genome myoviruses.

**Table 7:** **CoreGenes analysis indicates relationships among small genome myoviruses**

Reference genome (total # of gene products)	phiCD119 (53325 bp)	phiC2 (56,538 bp)	EJ-1 (42935 bp)	KC5a (38239 bp)	AQ113 (36566 bp)	Abouo (45552 bp)	Davies (58572 bp)	Jimmer1/2 (54312 bp)
phiCD119 (79 gp)	79 (100.00%)*							
phiC2 (82 gp)	40 (50.63%)	82 (100%)						
phiCD27 (75 gp)	29 (36.71%)	41 (50%)						
EJ-1 (73 gp)	12 (15.19%)	19 (23.17%)	73 (100%)					
KC5a (61 gp)	17 (21.52%)	20 (24.39%)	19 (26.03%)	61 (100%)				
AQ113 (56 gp)	16 (20.25%)	19 (23.17%)	18 (24.66%)	28 (45.9%)	56 (100%)			
Abouo (94 gp)	19 (24.05%)	25 (30.49%)	21 (28.77%)	19 (31.35%)	19 (33.93%)	94 (100%)		
Davies (94 gp)	20 (25.32%)	26 (31.71%)	21 (28.77%)	19 (31.35%)	19 (33.93%)	85 (90.43%)	94 (100%)	
Jimmer1/2 (102 gp)	19 (24.05%)	26 (31.71%)	20 (27.4%)	19 (31.35%)	18 (32.14%)	62 (65.96%)	65 (69.15%)	102 (100%)
Emery (102 gp)	7 (8.86%)	4 (4.88%)	5 (6.85%)	5 (8.2%)	3 (5.36%)	10 (10.64%)	12 (12.77%)	9 (8.82%)

*The number of genes in common is followed by a percentage in parenthesis that represents the% genes in common with the reference genome. Families are typically grouped based on a CoreGenes score of 40% or higher.

### Only one pham includes all five myoviruses, and very few phams are shared between unrelated *P. larvae*phages

Phages Emery and phiIBB_Pl23 are significantly different from each other and from the four similar *P. larvae* myoviruses, as is evident from the genome maps in Figure 3. However, Tables 4 and 5 demonstrate that some proteins encoded by these phages grouped into similar phams.

Pham 34 is the only pham in the database that included proteins from all five of the new myovirus *P. larvae* phages. These gene products are Abouo gp34, Davies gp34, Emery gp29, Jimmer1 gp36, and Jimmer2 gp36 and encode a bhlA/bacteriocin protein (Figure 3). No other gene products in the Phamerator database were grouped into this pham. The conserved domain in these proteins was DUF2762, a putative holin-like protein. When comparing amino acid sequences, these five proteins shared > 87% identity and an E-value less than 5 × 10^-43^.

**Figure 5:**
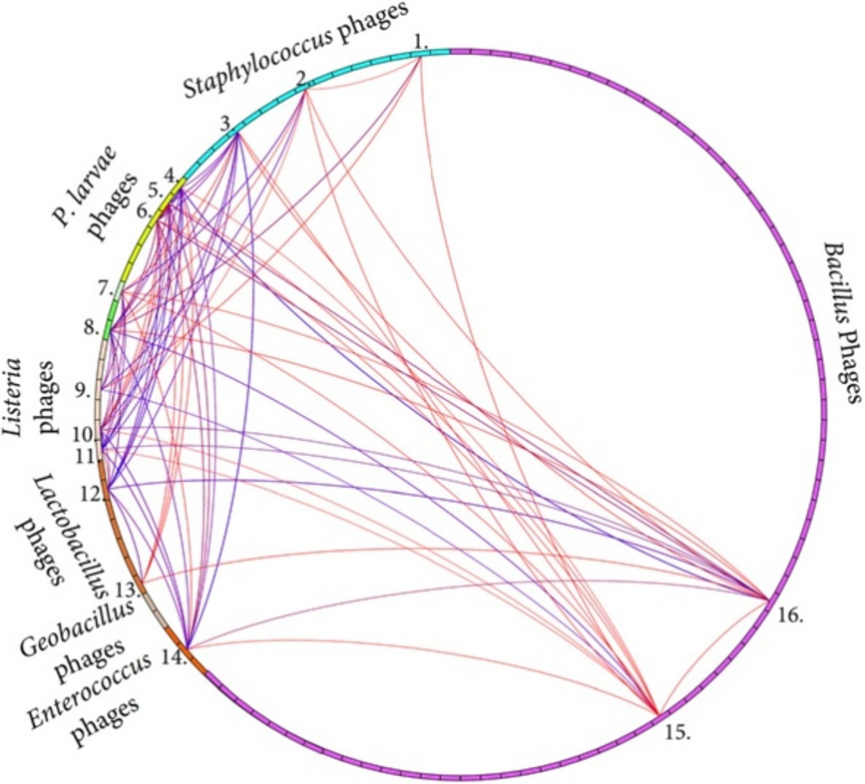
**Phamily circle connects 16 phages containing a related antirepressor gene.** The phamily circle of pham 951 connects all phages with genes encoding proteins in this pham. The phages containing gene products in pham 951 infect *Bacillus*, *Enterococcus*, *Lactobacillus*, *Listeria*, *Clostridium*, *Paenibacillus*, and *Staphylococcus* bacteria. The antirepressor gene is found in 16 different phages, including *Staphylococcus* phages (1) phiSLT (gp7), (2) PH15 (gp39), and (3) 55 (gp18); *Paenibacillus* phages (4) phiIBB_Pl23 (gp42), (5) Jimmer2 (gp19), (6) Jimmer1 (gp19), and (7) PG1 (gp28); *Clostridium* phage (8) phiC2 (gp52); *Listeria* phages (9) B054 (gp72), (10) A500 (gp36), and (11) A118 (gp46); *Lactobacillus* phages (12) phiAT3 (gp23), and (13) A2 (gp28); *Enterococcus* phage (14) phiEf11 (gp38); and *Bacillus* phages (15) SPBc2 (gp100) and (16) PM1 (gp26).

*P. larvae* siphovirus phiIBB_Pl23 contained only two proteins that shared a pham with any new myovirus *P. larvae* phages. The conserved domains in one gene product suggest it encodes a serine recombinase protein (Jimmer1 gp49, Jimmer2 gp49, phiIBB_Pl23 gp33) (Figure 3). The conserved domains in the other gene product suggest it encodes a phage antirepressor protein (Jimmer1 gp19, Jimmer2 gp19, phiIBB_Pl23 gp42) (Figure 3). Antirepressors from 13 other phages are also assigned to this pham (pham 951 in this database), including an antirepressor from *Paenibacillus glucanolyticus* phage PG1 gp28. A phamily circle links the 16 phages in the database containing a gene product in pham 951 (Figure 5).

The siphovirus *Paenibacillus glucanolyticus* phage PG1 also contained three gene products that shared a pham with *P. larvae* phages Abouo, Davies, Jimmer1, and Jimmer2. Pham 90 encoded an RNA polymerase sigma-70 factor and had 5 members: PG1 gp62, Abouo gp91, Davies gp91, Jimmer1 gp99, Jimmer2 gp99. Pham 75 encoded a recombination protein U and had 9 members, including PG1 gp42, Abouo gp73, Davies gp76, Jimmer1 gp83, and Jimmer2 gp83. Pham 78 encoded a dUTPase and had 13 members, including PG1 gp43, Abouo gp76, Davies gp79, Jimmer1 gp86, and Jimmer2 gp86.

### Four *P. larvae*phages contain duplicated genes

Phages Abouo and phiIBB_Pl23 did not contain any proteins that belonged to the same pham. However, gp52 and gp56 in phages Jimmer1 and Jimmer2 shared 52.1% identity (E-value is 1.39 × 10^-36^), belonged to pham 50, and encode an XRE family transcriptional regulator that contains a helix-turn-helix DNA binding domain. Additionally, gp62 and gp63 in phages Jimmer1 and Jimmer2 shared 40.3% amino acid identity (E-value is 8.32 × 10^-11^), belonged to pham 53, and contain an arc-like DNA-binding domain.

**Figure 6:**
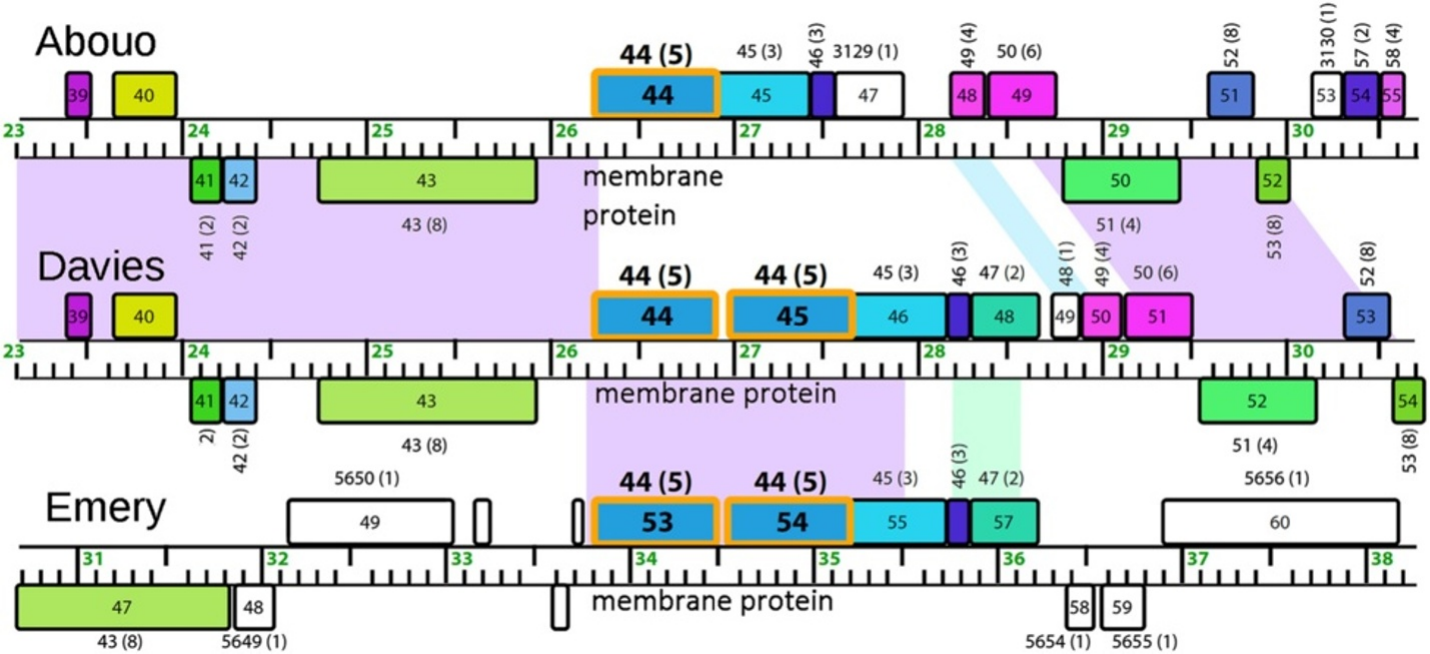
**Davies and Emery share a duplicated gene while Abouo has only one copy.** This genome map shows two gene products in both Davies and Emery that belong to pham 44 and encode a putative membrane protein. Davies gp44-47 and Emery gp53-56 are more similar to each other than they are to Abouo gp44-46. Gene product numbers are located inside the colored boxes. The numbers above each gene product indicate the pham number specific to this analysis and the (number of members in the pham). Gene products with the same color share a pham.

Davies gp44 and gp45 and Emery gp53 and gp54 belonged to pham 44 and encoded a putative membrane protein (Figure 6). Comparisons indicated that homologous proteins encoded on the two genomes were more similar than duplicated proteins encoded within one of the genomes. Davies gp44 and Emery gp53 shared 80.1% identity (E-value is 4.69 × 10^-107^), and Davies gp45 and Emery gp54 shared 82.9% identity (E-value is 3.20 × 10^-117^). However, Davies gp44 and gp45 shared 31.4% (E-value is 7.29 x 10^-28^) and Emery gp53 and gp54 shared 35.4% identity (E-value is 3.39 × 10^-34^). Abouo gp44 also belongs to pham 44 but the nucleotide sequence for this gene is different from the genes encoding the four gene products in Emery and Davies. Abouo gp44-46, Davies gp44-47, and Emery gp53-56 are identified in Figure 2A and 2B by the dark line indicating homology between Emery and Davies and the white gap between Abouo and Davies at the same location.

## Discussion

Prior to this report, nine *P. larvae* phages were described but were never analyzed collectively or grouped based on similar characteristics. Structural and morphological characteristics are the only published information for grouping the reported *P. larvae* phages to date. Therefore, for general comparison, *P. larvae* phages were identified as myovirus, elongated-capsid siphovirus, round-capsid siphovirus, or unknown siphovirus. The five *P. larvae* myoviruses characterized in this paper are structurally similar to previously isolated *P. larvae* myoviruses and may also be genetically similar. Since few phages infecting *P. larvae* have been sequenced, it is useful to compare structural similarity observed in electron micrographs. Now that sequencing data has been published for six *P. larvae* phage genomes and sequencing of others is sure to follow, genomic grouping will prevail and clusters will likely emerge as occurred with the mycobacteriophages [13].

The five myoviruses were isolated from three soil samples each from a separate location: Jimmer1 and Jimmer2 were isolated independently from the same sample [22], Emery and Abouo were isolated together, and Davies was isolated separately. *P. larvae* phage PBL2 was isolated from a different sample than BL2, yet all tests indicated no obvious structural or genetic differences between these phages [5]. Similar host properties and selective pressures can result in isolation of similar phages from different locations [13]. More *P. larvae* siphoviruses need to be sequenced before further correlations between genome and structural morphology can be drawn. As demonstrated in this work and by others, the sequence of the tape measure protein gene may be used to identify individual phages being studied if co-isolation occurs again in the future [38].

Bacteriophages are often unable to superinfect an existing lysogen if the entering and lysogenic phages are genetically similar [39]. The portion of the genome responsible for superinfection immunity has been determined for some phages [40]. Repressor genes involved in superinfection immunity have been characterized and are known to defend the prophage from premature lysis by silencing genes related to lysis [41]. This system does not work against phages that are always lytic or temperate phages that are not sensitive to the prophage repressor genes. Lysogens of Jimmer1, Jimmer2, and Davies displayed superinfection immunity when incubated with the same phage. Jimmer1 and Jimmer2 exhibited nearly identical sequences and were also immune to superinfections of each other.

The host specificity and correlating genome similarity between Jimmer1 and Jimmer2 (infect PL2) and Davies and Abouo (infect PL6) reflect common evolutionary ancestry. The high degree of similarity (over 80% average nucleotide identity) between the four phages may indicate that these phages infect a common host that has not yet been isolated or tested or that two phages recently switched hosts as is common in phages [42]. Jimmer1, Jimmer2, Davies, and Abouo likely coevolved.

Many bacteriophages contain genes that affect the virulence of the bacterial hosts. One toxin gene has been identified in phiIBB_Pl23 (gp26), and no toxin genes have been identified in the five *P. larvae* myoviruses. *P. larvae* phages Abouo, Davies, Jimmer1, Jimmer2, and phiIBB_Pl23 encode an ABC transporter-like protein. This was characterized as an extracellular protein produced by *P. larvae* [43], but it is not known how this protein is involved in host virulence. Future experiments involving the many putative XRE transcriptional regulators encoded by these phages may show a correlation with the virulence of *P. larvae*. Most of the transcriptional regulators found in the six *P. larvae* phages do not share phams with phages that infect any other bacterial host, indicating that these regulators are both phage- and host-specific. Two of these transcriptional regulators were duplicated in Jimmer1 and Jimmer2. The differences between these genes indicate that they are ohnologous and arose by gene duplication and subsequent divergence [44]. The duplicated genes in Emery and Davies are putative membrane proteins and likely evolved in a similar fashion. Abouo contains only one copy of this gene (Figure 6). BLAST hits for all six of the sequenced *P. larvae* phages show similarity to many proteins encoded by *Paenibacillus* and *Brevibacillus* bacteria. BLAST hits to these bacteria are not surprising because the genera *Paenibacillus* and *Brevibacillus* both belong to the family Paenibacillaceae and are closely related [45].

Analysis of large terminase protein phamilies revealed that Abouo, Davies, Jimmer1, and Jimmer2 likely use the headful packaging mechanism, while phiIBB_Pl23 likely has 3′ cohesive ends. Because of the stringent cutoff values required for inclusion in a pham, these results identify one way experimentally determined properties of a protein can be inferred on others sharing the same phamily.

Several gene products in *P. larvae* phages have similar functions but do not share phamilies. These include head morphogenesis, tape measure, baseplate, LysM peptidoglycan binding, peptidoglycan hydrolase, PBSX, and bacteriocin proteins. The conserved genes either diverged a long time ago or were acquired via convergent evolution. Additionally, the antirepressor protein in *P. larvae* phages phiIBB_Pl23, Jimmer1, Jimmer2 shares a pham with antirepressors from 13 other myoviruses and siphoviruses that infect host bacteria in the genera *Bacillus*, *Enterococcus*, *Geobacillus*, *Lactobacillus*, *Listeria*, *Paenibacillus*, and *Staphylococcus* (Figure 5). The presence of a similar antirepressor among phages of diverse Firmicute hosts may indicate the usefulness of the gene products and their associated conserved domains to regulate production of phage proteins within a diverse set of host bacteria. These data indicate that *P. larvae* phages have been subjected to multiple evolutionary pressures.

The head morphogenesis protein in phage Emery belongs to the SPP1 gp7 family and contains a ribosomal slippage site that is not found in *Bacillus* phage SPP1. Although two gene products are produced by the head morphogenesis gene in SPP1 that are 34 kDa and 28 kDa (compared to predicted proteins of 82.5 kDa and 58.9 kDa in Emery), the two SPP1 proteins are thought to be due to an alternative start site, not a frameshift caused by ribosomal slippage [46]. The lack of homology between protein sequences indicates these proteins further illustrates that Emery is not closely related to any other known bacteriophages.

Most of the putative encoded proteins in the *P. larvae* phages are not grouped into phams containing proteins from other phage types. These data indicate that most *P. larvae* phage genes are novel among currently identified genes of phages or bacteria. More than half of the *P. larvae* phage proteins have no identified conserved domains or putative functions, illustrating the diversity of bacteriophages and the vast number of unknown genes yet to be explored.

CoreGenes was previously used to verify current taxonomic relationships between phages in the *Podoviridae* [36] and *Myoviridae* families [37]. It was also used analyze other "dwarf" myoviruses and group them based on the similarity of core genes [47]. Analysis of core genes and shared phams indicates that *P. larvae* phages Abouo, Davies, Jimmer1, and Jimmer2 are distantly related to phages in the phiCD119likevirus family as well as phages EJ-1, KC5a, and AQ113. Because proteins grouped into similar phams are phylogenetically related, these proteins likely share a common ancestry. The structural similarities between phages Abouo, Davies, Jimmer1, Jimmer2, phiC2, KC5a, AQ113, and EJ-1 may correlate with their genetic similarities because the conserved core genes include the structural module of each genome. However, the current accepted threshold of 40% for a sufficiently strong CoreGenes percentage prevents any of these phages from being grouped taxonomically (except perhaps KC5a and AQ113, which is not within the scope of this paper). The differences in genome lengths may also prevent the formation of a taxonomic family of these phages as CoreGenes reflects the percentage based on the number of genes, which means that genome length differences and subsequent differences in total gene numbers within a genome can influence the score.

The results of the CoreGenes analysis indicate that *P. larvae* phages Abouo, Davies, Jimmer1, and Jimmer2 are related phylogenetically. They are also distantly related to phiC2, KC5a, AQ113, and EJ-1 which infect other bacterial hosts. This relationship indicates that these four phages are the closest known phylogenetic relatives to these four *P. larvae* phages. The conservation of primarily structural genes among the eight small genome myoviruses may indicate that the phages adapted to maintain infectivity as their bacterial hosts diverged, but retained ancestral structural genes that were under less selective pressure.

Conserved genes between different phages may indicate important genes. The bacterial hosts PL2 and PL6 are similar (according to the 16S rRNA sequences and physical properties), and similar BhlA/bacteriocin genes such as found in the shared pham of Jimmer1, Jimmer2, Emery, Abouo, and Davies (pham 34) can likely be used to lyse the bacterial host. It is interesting to note that the only two genes shared between phiIBB_Pl23 and any other *P. larvae* phage encode a serine recombinase and an antirepressor, shared with the PL2 phages Jimmer1 and Jimmer2. This correlation may indicate similar host interactions, as these genes help regulate the lytic and lysogenic cycles. The PL6 phages do not contain any antirepressor gene products belonging to this pham. Although *P. larvae* phages Emery and phiIBB_Pl23 do not show significant genetic relatedness to any other sequenced phages, similar genes and phages will likely be discovered in the future. The six newly sequenced genomes of the *P. larvae* phages compared in this report are an initial foundation for future studies.

## Conclusions

This first comparison of *P. larvae* phage genomes provides insight into the genus *Paenibacillus* and the important honeybee bacterial pathogen, *P. larvae*. Although six *P. larvae* phages show some relatedness to phages that infect other Firmicute bacteria, most *P. larvae* phage genes do not share phams with non-*P. larvae* phages and many gene products still have unknown functions. Efforts to characterize these gene products and to isolate, sequence, and analyze new *P. larvae* phages will help us better understand the genetics of these phages and their bacterial host.

## Methods

### Identification of field isolates

*Paenibacillus larvae* spores were extracted from local honey samples using the process described by Hornitzky [48]. Pelleted spores were streaked on PLA [49] plates that contained nalidixic and pipemidic acid and plates were incubated for 48–72 hours at 37°C. Colonies were streaked to purity on PLA plates. Isolates were tested with hydrogen peroxide for the presence of the catalase enzyme [50] and were tested by gram stain [45, 51].

A single colony from each bacterial field isolate was boiled at 98°C for five minutes, and 3 μL of the lysate was used as a PCR template. The 16S rRNA gene region was amplified using universal primers 27 F and 907R [52], and the standard protocol for Taq DNA polymerase (New England Biolabs). Following PCR, amplicon size was checked by agarose gel electrophoresis. Samples producing a ~1 kb band were submitted for BigDye (Applied Biosystems, Life Technologies) sequencing to the BYU DNA Sequencing Center. Resulting 16S sequences were analyzed using BLAST [19].

### Superinfection of *P. larvae*lysogens with phage

Phages described by Sheflo et al. [22] were used in lysogenic superinfection studies using a protocol adapted from [53]. Some agar was removed from the center of an isolated plaque, streaked out on an LB plate, and incubated at 37°C for 24 hours to allow any lysogens to grow. One colony was removed, incubated at 37°C in 1 ml of LB broth for two hours, and then plated using the method described above. When the top agar was solid, 5 μL of each phage lysate was placed on the plate. The plate was incubated agar side down at 37°C for 24 hours. Clearing under the spots indicated superinfection had occurred, while no clearing indicated that the lysogenic bacteria were immune to superinfection.

### Electron microscopy of *P. larvae*phages

Electron microscopy was performed at Brigham Young University in the Life Sciences Microscopy Lab using an FEI Tecnai 12 Spirit transmission electron microscope. To prepare the samples for imaging, 20 μl of high-titer phage lysate was placed on a 200-mesh copper carbon type-B electron microscope grid for one minute. The lysate was wicked away and the grids were stained for two minutes using 2% phosphotungstic acid (pH = 7). Residual liquid was wicked away and the grid was allowed to dry before being imaged. Phage structures in electron micrographs were measured using ImageJ [54]. The average and standard deviation for each measurement was calculated from a minimum of three separate measurements.

### Genomic comparison of sequenced phages

The DNA sequences for the six sequenced *P. larvae* phages were downloaded from GenBank using reported accession numbers [21, 22]. Dotplots of nucleic acid and protein sequences were generated using Gepard [55] and then compared. ClustalW [56] was used to calculate Average Nucleotide Identity (ANI) percentages comparing each of the *P. larvae* phage genomes. The online tool FrameD [25] was used to search for frameshift mutations. Core genes were identified using the program CoreGenes 3.5 [57, 58] with the default BLASTP threshold of 75.

Phages genes were analyzed using Phamerator [17], an open-source program (GNU general public license) designed to compare phage genes and genomes. For this study, Phamerator was adapted and stored in a GitHub repository ( http://github.com/byuphamerator/phamerator-dev) separate from the original version. Phamerator uses BLASTP [19] and ClustalO [20] to compare each protein encoded by the genes in the database. E-values and percent identity scores are used to sort proteins into groups referred to as phamilies (phams) based on user-defined cutoffs for each score. Conserved domains in each protein are then identified. The Phamerator database used in this study was populated with 71 *Bacillus* phages, one *Clostridium* phage, 3 *Enterococcus* phages, 2 *Geobacillus* phages, 7 *Lactobacillus* phages, 6 *Listeria* phages, 6 *P. larvae* phages, one *Paenibacillus glucanolyticus* phage, 15 *Staphylococcus* phages, and one *Streptococcus* phage. The non-*Bacillus* phages were included in the database because proteins from these phages appeared in low E-value (<0.0001) BLAST hits for *P. larvae* phage proteins. In this Phamerator database, genes with E-values smaller than 1 × 10^-50^ or greater than 32.5% identity with at least one other protein were grouped into phams. These parameters were identical to those used by Cresawn et al. [17]. Conserved domains in proteins were identified using RPS-BLAST [59] to search the Conserved Domain Database (CDD) released by NCBI on 21 March 2013 [18]. The 100 published phages in the Phamerator database are listed below with the respective bacterial hosts and accession numbers. The Bacillus_Draft database can be accessed through Phamerator (see http://phagesdb.org/Phamerator/faq/). The Phamerator database for this study (bphage5) is available at http://phagehunters.byu.edu/BeeProject.aspx.

*Bacillus* phages vB_BceM_Bc431v3 (*B. cereus*) [NC_020873], 0305phi8-36 (*B. thuringiensis*) [NC_009760], 250 (*B. cereus*) [GU229986], Andromeda (*B. pumilus*) [KC330684], AP50 (*B. anthracis*) [NC_011523], B103 (*B. subtilis*) [NC_004165], B4 (*B. cereus*) [JN790865], B5S (*B. cereus*) [JN797796], Bam35c (*B. thuringiensis*) [NC_005258], Basilisk (*B. cereus*) [KC595511], Bastille (*B. cereus*) [NC_018856], BCD7 (*B. cereus)* [NC_019515], BceA1 (*B. cereus*) [HE614282], BCJA1c (*B. clarkii*) [NC_006557], BCP78 (*B. cereus*) [NC_018860], BCU4 (*B. cereus*) [JN797798], BMBtp2 (*B. thuringiensis*) [JX887877], BPS13 (*B. cereus*) [NC_018857], BtCS33 (*B. thuringiensis*) [NC_018085], CAM003 (*B. thuringiensis*) [NC_024216.1], Cherry (*B. anthracis*) [NC_007457], Curly (*B. pumilus*) [KC330679], Eoghan (*B. pumilus*) [KC330680], Evoli, (*B. thuringiensis*) [NC_024207.1], Fah (*B. anthracis*) [NC_007814], Finn (*B. pumilus*) [KC330683], G (*B. megaterium*) [JN638751], GA-1 (*Bacillus sp.*) [NC_002649], Gir1 (*Bacillus sp.*) [Bacillus_Draft], Gamma 51 (*B. cereus*) [DQ222853], Gamma 53 (*B. anthracis*) [DQ222855], Gamma isolate d’Herelle (*B. cereus*) [DQ289556], Gemini (*B. pumilus*) [KC330681], GIL16c (*B. thuringiensis*) [NC_006945], Hakuna (*B. thuringiensis*) [NC_024213.1], Hoody T (*B. thuringiensis*) [NC_024205], IEBH (*B. thuringiensis*) [NC_011167], JL (*B. cereus*) [KC595512], JPB9 (*B. thuringiensis*) [Bacillus_Draft], Megatron (*B thuringiensis*) [NC_024211.1], MG-B1 (*B. weihenstephanensis*) [NC_021336], Nf (*B. subtilis*) [EU622808], Pappano (*B. pumilus*) [Bacillus_Draft], PBC1 (*B. cereus*) [NC_017976], Pegasus (*Bacillus sp.*) [bacillus.phagesdb.org], pGIL01 (*B. thuringiensis*) [AJ536073], phi105 (*B. subtilis*) [NC_004167], phi29 (*B. Subtilis*) [NC_011048], phiAGATE (*B. pumilus*) [JX238501], phiNIT1 (*B. subtilis*) [NC_021856], phIS3501 (*B. thuringiensis*) [NC_019502], Pleiades (*B. pumilus*) [Bacillus_Draft], PM1 (*B. subtilis*) [NC_020883], Polaris (*B. pumilus*) [Bacillus_Draft], PZA (*B. subtilis*) [M11813], Riley (*B. thuringiensis*) [KJ489402], Shanette (*B. cereus*) [KC595513], SP10 (*B. subtilis*) [NC_019487], SPbeta (SPBc2) (*B. subtilis*) [NC_001884], SPO1 (*B. subtilis*) [NC_011421], SPP1 (*B. Subtilis*) [NC_004166], Stitch (*Bacillus sp.*) [Bacillus_Draft], Taylor (*B. pumilus*) [KC330682], TP21-L (*B. cereus*) [NC_011645], Troll (*B. thuringiensis*) [KF208639], W.Ph. (*B. cereus*) [NC_016563], WBeta (*B. cereus*) [NC_007734], Wip1 (*B. anthracis*) [KF188458], phBC6A51 (*B. cereus*) [NC_004820], phBC6A52 (*B. cereus*) [NC_004821], *Bacillus* virus 1 (*Bacillus. sp. 6 k512*) [NC_009737]; *Clostridium* phage phiC2 (*C. difficile*) [NC_009231]; *Enterococcus* phages phiEf11 (*E. faecalis*) [NC_013696], phiEF24C (*E. faecalis*) [NC_009904], phiFL3A (*E. faecalis*) [NC_013648]; *Geobacillus* phages GBSV1 (*Geobacillus sp. 6 k51*) [NC_008376], E2 (*Geobacillus*) [NC_009552]; *Lactobacillus* phages A2 (*L. casei*) [NC_004112], KC5a (*L. gasseri*) [NC_007924], Lb338-1 (*L. paracasei*) [NC_012530], Lc-Nu (*L. rhamnosus*) [NC_007501], LP65 (*L. plantarum*) [NC_006565], phiAT3 (*L. casei)* [NC_005893], phig1e (*Lactobacillus*) [NC_004305]; *Listeria* phages P100 (*L. monocytogenes*) [DQ004855], A118 (*L. monocytogenes*) [NC_003216], A500 (*L. monocytogenes*) [NC_009810], A511 (*L. monocytogenes*) [NC_009811], B054 (*L. monocytogenes*) [NC_009813], P40 (*L. monocytogenes*) [NC_011308]; *Paenibacillus* phages Abouo (*P. larvae*) [KC595517], Davies (*P. larvae*) [KC595518], Emery (*P. larvae*) [KC595516], Jimmer1 (*P. larvae*) [KC595515], Jimmer2 (*P. larvae*) [KC595514], PG1(*P. glucanolyticus*) [HQ332138], phiIBB_Pl23 (*P. larvae*) [KF010834]; *Staphylococcus* phages phi 12 (*S. aureus*) [AF424782], 37 (*S. aureus*) [NC_007055], 3A (*S. aureus*) [NC_007053], 47 (*S. aureus*) [NC_007054], 55 (*S. aureus*) [NC_007060], 88 (*S. aureus*) [NC_007063], G1 (*S. aureus*) [NC_007066], K (*S. aureus*) [NC_005880], PH15 (*S. epidermidis*) [NC_008723], phiSLT (*S. aureus*) [NC_002661], SA11-v (*S. aureus*) [NC_019511], SpaA1 (*S. pasteuri*) [NC_018277], Twort (*S. aureus*) [NC_007021], X2 (*S. aureus*) [NC_007065], tp310-2 (*S. aureus*) [NC_009762]; *Streptococcus* prophage EJ-1 (*S. pneumoniae*) [NC_005294].The neighbor-joining phylogenetic tree (Figure 4) was generated using ClustalO. The phage large terminase proteins included in this tree are listed below with their respective accession numbers.

*Bacillus* phages 0305phi8-36 gp117 [YP_001429607.1], 250 gp5 [ADB28373.1], Andromeda gp9 [YP_007517474.1], B4 gp4 [YP_006908233.1], B5S gp4 [AEW47238.1], Basilisk gp3 [AGR46580.1], Bastille gp267 [AEQ34197.1], BCD7 gp104 [YP_007005955.1], BceA1 gp4 [CCE73839.1], BCJA1c gp34 [YP_164412.1], BCP78 gp12 [YP_006907847.1], BCU4 gp5 [AEW47511.1], BMBtp2 gp40 [YP_007236398.1], BPS13 gp6 [YP_006907565.1], BtCS33 gp2 [YP_006488672.1], Cherry gp2 [YP_338134.1], Curly gp9 [YP_007517553.1], Eoghan gp9 [YP_007517399.1], Fah gp2 [YP_512312.1], Finn gp9 [YP_007517630.1], G gp1 [YP_009015312.1], Gamma51 gp2 [ABA46445.1], Gamma isolate d’Herelle gp2 [ABC40454.1], Gemini gp9 [AGE60848.1], IEBH gp49 [YP_002154374.1], JL gp4 [AGR46722.1], PBC1 gp2 [YP_006383455.1], phBC6A52 gp2583 [NP_852588.1], phiAGATE gp196 [YP_007349220.1], phiNIT1 gp79 [YP_008318309.1], phIS3501 gp23 [YP_007004362.1], Shanette gp4 [AGR46934.1], SP10 gp119 [YP_007003376.1], SPO1 gp2.11 [YP_002300330.1], Taylor gp9 [AGE60927.1], Troll gp61 [YP_008430845.1], vB_BceM_Bc431v3 gp10 [YP_007676908.1], W.Ph. gp3 [YP_004957018.1], WBeta gp2 [YP_459966.1], and *e* virus 1 gp18 [YP_001425601.1]; *Clostridium* phage phiC2 gp2 [YP_001110720.1], *Enterococcus* phages phiEf11 gp2 [YP_003358792.1], phiEF24C gp5 [YP_001504114.1], and phiFL3A gp43 [YP_003347605.1]; *Geobacillus* phages GBSV1 gp17 [YP_764473.1] and E2 gp2 [YP_001285808.1]; *Lactobacillus* phages A2 gp2 [NP_680484.1], KC5a gp35 [YP_529870.1], Lb338-1 gp89 [YP_002790768.1], Lc-Nu gp2 [YP_358760.1], LP65 gp113 [YP_164748.1], phiAT3 gp2 [YP_025027.1], and phig1e gp37 [NP_695170.1]; *Listeria* phages A118 gp2 [NP_463463.1], A500 gp2 [YP_001468388.1], A511 gp5 [YP_001468454.1], B054 gp2 [YP_001468706.1], P100 gp5 [AAY53308.1], and P40 gp2 [YP_002261418.1]; *Paenibacillus* phages Abouo gp2 [AGR47449.1], Davies gp2 [YP_008858637.1], Emery gp3 [AGR47349.1], Jimmer1 gp2 [AGR47249.1], Jimmer2 gp2 [AGR47149.1], and phiIBB_Pl23 gp2 [YP_008320338.1]; *Staphylococcus* phages PH15 gp2 [YP_950664.1], phi 12 gp30 [NP_803336.1], phiSLT gp39 [BAB21732.1], SpaA1 gp4 [YP_006560692.1], and tp310-2 gp45 [ABS87507.1]; *Streptococcus* phage EJ-1 gp39 [NP_945278.1]; and *Mycobacterium* phage Nigel gp4 [YP_002003843.1].

### Availability of supporting data

The data set supporting the results of this article is included within the article (and its Additional files). The same files are also available at http://phagehunters.byu.edu/BeeProject.aspx.

## Electronic supplementary material

Additional file 1: **Phamerator database containing the 113 phage genomes.** (ZIP 7 MB)

Additional file 2: **Spreadsheet exported from the Phamerator database to report all phage gene products in the database, the phams to which the gene products are assigned, and the conserved domains found in gene products in those phams.** (XLSX 1 MB)
